# Dexmedetomidine regulates sleep rhythm and alleviates neuroinflammation in rats under high-altitude hypoxia

**DOI:** 10.1007/s13105-025-01127-1

**Published:** 2025-11-12

**Authors:** Shanshan Liu, Long Feng, Haining Yang, Yi Liu, Wen Sun, Yongzhe Liu, Longhe Xu

**Affiliations:** 1 Department of Anesthesiology, Graduate School of Jinzhou Medical University, Jinzhou, 121001 China; 2 Department of Anesthesiology, 3rd Medical Centre of the Chinese PLA General Hospital, No. 69, Yongding Road, Haidian District, Beijing, 100039 China; 3 Department of Anesthesiology, Hainan Medical Centre of the Chinese PLA General Hospital, Beijing, 100039 China; 4 Department of Emergency, Qinghai Armed Police Corps Hospital, Xining, 810000 China

**Keywords:** High-altitude hypoxia, Sleep rhythm, Neuroinflammation, Anxiety-like behavior, Dexmedetomidine, Rat model

## Abstract

High-altitude hypoxia (HH) significantly affects mammalian physiological functions, especially sleep rhythms, anxiety-like behavior, and neuroinflammation. In this study, adult male Sprague-Dawley rats were randomly assigned to five groups: Control, HH, HH with low-dose dexmedetomidine (Dex), HH with high-dose Dex, and Control with Dex. Rats were exposed to a simulated 6000-meter altitude for 7 days. Behavioral tests, enzyme-linked immunosorbent assay (ELISA), reverse transcription quantitative PCR (RT-qPCR), and Western blotting were used to assess hormone levels, gene/protein expression, and inflammatory markers. HH exposure elevated hypothalamic serotonin (5-hydroxytryptamine, 5-HT) and reduced melatonin levels. The sleep rhythm-related gene Timeless was downregulated, while Homer1 was upregulated at both mRNA and protein levels. Knockdown of Timeless or overexpression of Homer1 resulted in a significant increase in hypothalamic 5-HT levels and a marked decrease in melatonin levels. HH induced enhanced anxiety-like behaviors and reduced spontaneous activity, accompanied by elevated levels of L-1β, IL-6, and TNF-α in both serum and hypothalamus. Inhibition of the TLR4-MyD88-NFκB pathway significantly alleviated both hormonal disturbances and anxiety-like behaviors in HH rats.Dex treatment, especially at high doses, suppressed inflammatory responses, normalized hormone levels, restored sleep gene expression, and improved behavioral outcomes. These results indicate that dex mitigates HH-induced sleep and inflammatory disturbances, highlighting its therapeutic potential for high-altitude neurological dysfunction.

**Graphical Abstract:**

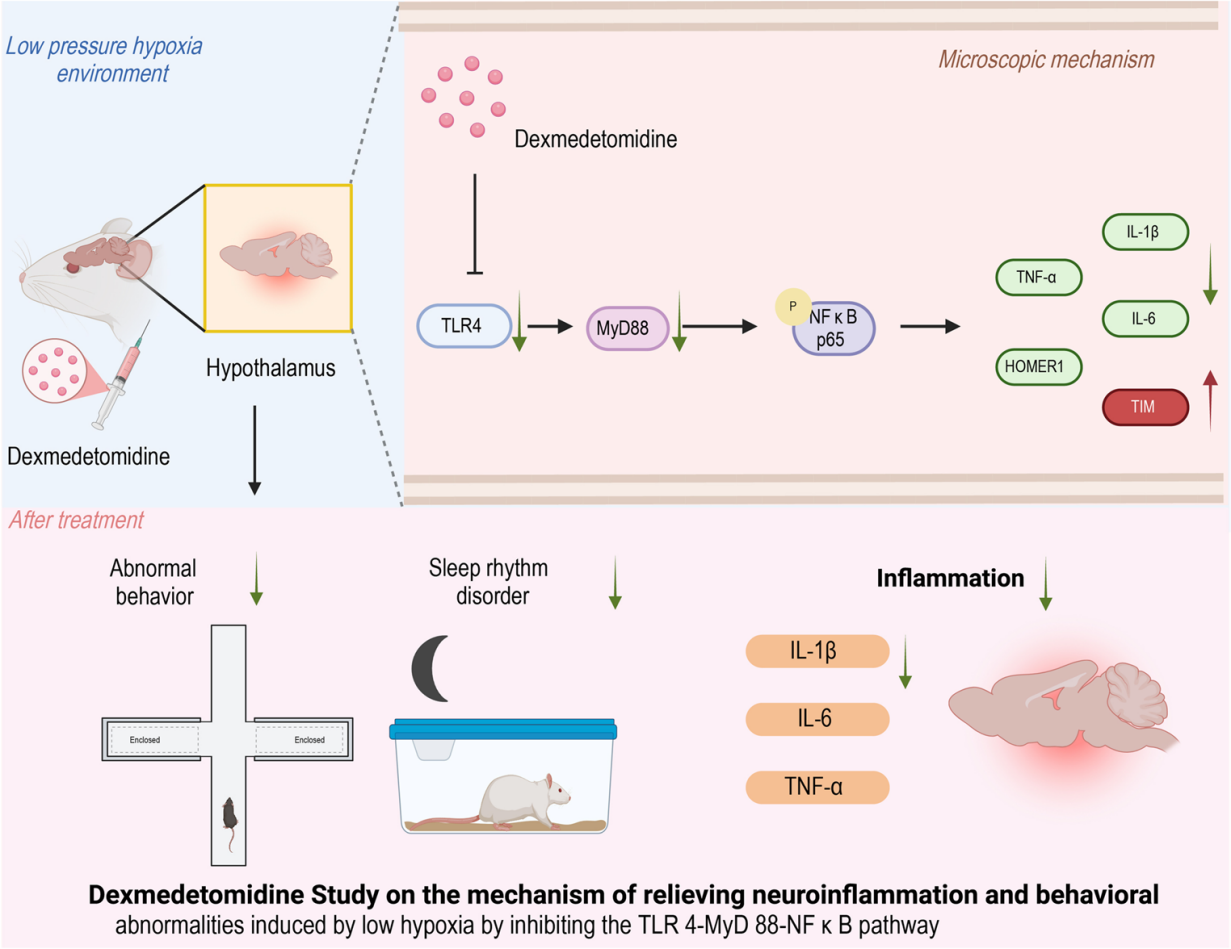

**Supplementary Information:**

The online version contains supplementary material available at 10.1007/s13105-025-01127-1.

## Introduction

High-altitude hypoxia (HH) refers to the condition in which reduced oxygen concentrations in the atmosphere at higher altitudes lead to insufficient oxygen supply to the organism. Prolonged exposure to HH triggers a series of adaptive mechanisms in the body to cope with the hypoxic challenge, such as increasing red blood cell count, enhancing respiratory rate, and accelerating heart rate, in order to maintain oxygen supply to tissues and organs [1]. However, these adaptive responses may also have negative consequences, particularly in the regulation of the nervous system [2, 3]. In recent years, research on the effects of HH on brain function has gradually increased, especially regarding sleep rhythms, emotional regulation, and cognitive functions [4, 5]. HH not only alters the physiological functions of the central nervous system but may also lead to behavioral changes such as anxiety and depression, which have profound impacts on residents living in high-altitude areas and individuals working in high-altitude conditions [6, 7]. Therefore, exploring the effects of HH on the nervous system, particularly its influence on sleep rhythms, has become an important topic in neurobiological research.

Sleep rhythm refers to the sleep-wake patterns in the Human body over a 24-hour cycle, primarily regulated by the brain’s biological clock. Studies have shown that the regulation of sleep rhythm is influenced by various factors, particularly the roles of neurotransmitters and hormones [8–10]. 5-hydroxytryptamine (5-HT), a neurotransmitter that plays a crucial role in the central nervous system, is essential for regulating different stages of sleep [11, 12]. Melatonin, a hormone secreted by the pineal gland, primarily regulates the circadian rhythm, influencing the onset and maintenance of sleep. HH is known to disrupt the levels and biological functions of these key molecules, thereby affecting sleep patterns [13]. Therefore, exploring the regulatory effects of HH on molecules such as 5-HT and melatonin is of significant importance for a deeper understanding of the mechanisms underlying changes in sleep rhythm.

HH not only affects sleep rhythm but also leads to other functional changes in the nervous system [14, 15]. The hypothalamus is a key brain region involved in the regulation of sleep, mood, and autonomic behavior and is particularly susceptible to hypoxia [16]. Hypoxia activates a series of signaling pathways related to neuroinflammation, resulting in neuronal damage, neurofunctional impairment, and behavioral changes. Recent studies have shown that hypoxia-induced neuroinflammation may promote neuronal injury by upregulating pro-inflammatory factors such as including Interleukin-1 beta (IL-1β), Interleukin-6 (IL-6), and Tumor Necrosis Factor-alpha (TNF-α) [17, 18]. These inflammatory responses not only affect the structure and function of the nervous system but may also be closely associated with the development of mood disorders such as anxiety and depression. Therefore, investigating the neuroinflammatory responses triggered by hypoxia and its effects on behavior provides new directions for finding effective interventions.

Previous studies on HH-induced neurological dysfunction have primarily focused on hormonal alterations and behavioral changes, while the role of inflammatory pathways in circadian regulation remains poorly understood. Although melatonin and benzodiazepines are widely used to treat sleep disorders, their efficacy under hypoxic stress is inconsistent, and their mechanisms in this context remain unclear [19]. Reviews suggest that melatonin may exert antioxidant, anti-inflammatory, and neuroprotective effects under hypoxia [20], yet direct evidence linking melatonin to circadian-inflammation interactions in hypoxic conditions is lacking. Dexmedetomidine (Dex), a selective α2-adrenergic receptor agonist commonly used in clinical anesthesia and sedation [21, 22], has shown promising neuroprotective and anti-inflammatory effects in recent studies [23–25]. Dex promotes NREM sleep and stabilizes circadian rhythms via activation of SCN^VIP neurons [26] and modulates neuroinflammation and anxiety-like behavior by targeting the TLR4-MyD88-NF-κB signaling pathway [27, 28]. Additional research confirms its ability to mitigate neuronal injury and improve neurobehavioral outcomes under hypoxia [29–31].

This study investigates the disruption of sleep rhythms and induction of neuroinflammation under HH, and examines the modulatory effects of Dex on these pathological changes. A rat model of simulated HH was used to assess hypothalamic levels of 5-hydroxytryptamine and melatonin, the expression patterns of circadian rhythm-related genes, activation of inflammatory signaling pathways, and associated behavioral alterations. The results provide mechanistic insight into the interplay between hypoxia-induced circadian dysregulation and neuroinflammation, and identify Dex as a potential pharmacological strategy for the prevention of hypoxia-related neurological dysfunction.

## Materials and methods

### Animal experiment

Adult male Sprague-Dawley rats (weight 80–120 g) were purchased from SPF (Beijing) Biotechnology Co., Ltd. and randomly assigned to eight experimental groups (Fig. S1): Control group (Ctrl), HH group (HH), HH + Low-dose Dex group (HH + D1), HH + High-dose Dex group (HH + D2), Control + Low-dose Dex group (Ctrl + D2), sh-Timeless group (Timeless knockdown), sh-TLR4 group (TLR4 knockdown), and oe-Homer1 group (Homer1 overexpression). In the Control group (Ctrl), rats were not exposed to the HH and did not receive Dex injections. In the HH group, a multifunctional environmental simulation chamber was used to simulate conditions equivalent to an altitude of 6000 m. Animals were acclimated in the chamber for three days, after which the oxygen concentration was gradually reduced and maintained for seven consecutive days. In the HH + D1 and HH + D2 groups, rats received intraperitoneal injections of Dex at 50 µg/kg/day [31] and 100 µg/kg/day [29], respectively, at 08:00 during hypoxic exposure. In the Ctrl + D2 group, rats were Maintained under normoxia and received 50 µg/kg/day Dex at the same time [31].

Gene modulation was performed via tail vein injection of the corresponding viral vectors 7 days prior to the experiment. Rats in the sh-Timeless, sh-Tlr4, and oe-Homer1 groups received lentiviral particles (GenePharma; titer ≥ 1 × 10^8^ TU/mL) to achieve knockdown or overexpression of the target genes. An additional group was injected with AAV1 virus (Hanbio Biotechnology, Shanghai, China) at a dose of 13 v.g/mL. Following injection, the rats were allowed to acclimate under standard conditions for 7 days, and were then exposed to a hypobaric hypoxia environment simulating 6000 m altitude for another 7 days, without Dex intervention. After the exposure, all samples were collected for subsequent analysis. The shRNA sequence targeting Timeless was 5’-CCCTGATTTGTGTTTCATATT-3’, constructed into the LV3 lentiviral vector; the shRNA sequence targeting Tlr4 was 5’-GCCAATCCTAAGAATGCTATA-3’; and the Homer1 overexpression vector (LV5) contained the full-length rat Homer1 cDNA. All lentiviral vectors were synthesized, packaged, and purified by GenePharma. The safety of this dose range is further supported by previous studies using 1 mg/kg Dex in adult rats [32].

During the experiment, all animals were housed at the Experimental Animal Center of the Chinese Academy of Military Sciences and had free access to food and water. The Light cycle was set to 12 h of light per day. After the intervention, all animals underwent behavioral testing and were immediately euthanized. The experiment adhered to the Guide for the Care and Use of Laboratory Animals (NIH Publications No. 8023) and the ARRIVE guidelines by NC3Rs. All possible measures were taken to minimize animal suffering.

### Open field test (OFT)

The OFT was conducted between 08:00 and 11:00 in a square arena (1 m²) divided into 25 equal grids. At the start of each trial, the rat was placed in a corner of the arena, and behavior was recorded for 10 min. The recorded parameters included: spontaneous activity: total distance traveled and number of rearing events. Anxiety-like behavior: number of entries into the central area, duration of time spent in the central area, and distance traveled within the central area.

### Elevated plus maze (EPM)

The EPM test was conducted between 08:00 and 11:00. The maze consisted of two open arms (5.90 cm wide, 29.60 cm long) and two closed arms of the same size, enclosed by 15.00 cm high walls (0.3 cm thick), with a central platform measuring 5.80 × 5.80 cm. The entire apparatus was elevated 39.30 cm above the floor. Each rat was placed on the central platform under dim lighting (~ 12 lx) in a quiet environment and allowed to explore freely for 5 min. Behavior was recorded and analyzed using a video tracking system (V3.0, Harvard Apparatus) and Smart software (Panlab, Harvard Apparatus). For each animal, the number of entries into the open arms and the time spent in open arms were quantified [5].

### Sample Preparation

Following anesthesia with sodium pentobarbital (3%, 45 mg/kg, intraperitoneally) [33], the thoracic cavity was opened to fully expose the heart. Blood was drawn from the apex of the heart and placed into a heparinized tube. The sample was then centrifuged at 3500 rpm for 10 min, and the supernatant was collected as plasma.

Tissue Homogenate for Western Blot (WB): RIPA buffer and protease inhibitors were added to the tissue at a ratio of 3 µL per milligram of tissue. The tissue was then homogenized using an ultrasonic cell disruptor, followed by centrifugation at 12,000 rpm for 15 min at 4 °C. The supernatant was collected for protein analysis by WB.

Tissue Homogenate for Enzyme-Linked Immunosorbent Assay (ELISA): The hypothalamus was homogenized in physiological saline at a ratio of 10 µL per milligram of fresh tissue. The tissue was ground using a tissue homogenizer and then centrifuged at 20,000 rpm for 15 min at 4 °C. The supernatant was collected for ELISA detection.

### Hypothalamic protein detection (WB)

Protein concentration was quantified using the BCA method (Thermo Fisher Scientific, 23227). Proteins were separated using 10% SDS-PAGE gel (Bio-Rad, 4561093), with 15 µg of total protein loaded per well. Electrophoresis was performed at a constant voltage of 90 V, followed by protein transfer to a PVDF membrane at 80 V for 2 h. The membrane was blocked with 5% BSA at room temperature for 1.5 h. The membrane was then incubated with primary antibodies: rabbit polyclonal TLR4 (Abcam, 1:1000, ab13556), TIM (Abcam, 1:1000, ab47637), HOMER1 (Synaptic Systems, 160 003, 1:2000), MYD88 (Cell Signaling Technology, 4283, 1:1000), p-NF-κB p65 (Cell Signaling Technology, 3033, 1:1000), NF-κB p65 (Proteintech, 10745-1-AP, 1:2000), GAPDH (Abcam, 1:1000, ab8245) at 4 °C for 15 h. Afterward, the membrane was incubated with secondary HRP-conjugated goat anti-rabbit IgG (CST, 1:5000) at room temperature for 2 h. Detection was performed using ECL chemiluminescent reagents (Thermo Fisher Scientific, 32106), and the signals were captured and analyzed with the GE ImageQuant LAS 4000.

### Reverse transcription quantitative PCR (RT-qPCR)

RNA from the hypothalamus was extracted using the Trizol method (Invitrogen, 15596026). After RNA quality control, reverse transcription was performed using the ABScript Master Mix (ABclonal, RK20403). The RT-qPCR reaction system included 10 µL of ABScript Master Mix, 0.4 µL of forward and reverse primers (10 µM), 0.4 µL of cDNA, and 9.2 µL of RNase-free water. The cycling conditions were as follows: 95 °C for 5 min for pre-denaturation, followed by 40 cycles of 95 °C for 15 s and 60 °C for 30 s. Primer information is provided in Table S1. Data analysis was performed using the ΔΔCT method.

### Elisa

ELISA kits were used to measure the levels of IL-1β, IL-6, and TNF-α in the hypothalamus (IL-1β ELISA Kit, R&D Systems, DY501; IL-6 ELISA Kit, R&D Systems, DY506; TNF-α ELISA Kit, R&D Systems, DY510). Samples or standards were added to an ELISA plate and incubated at 37 °C for 2 h, followed by washing. HRP-conjugated antibody and working solution were added, and incubation continued at 37 °C for another 30 min. After adding the chemiluminescent substrate, the optical density (OD) value was read using a microplate reader.

###  Immunofluorescence staining

Immunofluorescence staining was performed to detect IBA-1 expression in microglia within rat brain tissue. Briefly, 8 μm cryosections were fixed in cold methanol/acetone (1:1) at − 20 ℃ for 10 min. After PBS washing, sections were blocked with 3% bovine serum albumin (BSA) in PBS at room temperature and then incubated at 4℃ for 60 min with primary antibodies against IL-6 (1:100, sc-28343, Santa Cruz, USA) and IBA-1 (1:100, DF6442, Affinity Biosciences, China). Following primary incubation, sections were treated with secondary antibodies—goat anti-rabbit IgG conjugated to FITC (1:400, ab6717, Abcam) and goat anti-mouse IgG conjugated to Cy3 (1:400, M30010, Thermo Fisher)—for 30 min at 37 ℃. Nuclei were counterstained with DAPI in the dark. After final PBS washes, sections were mounted for imaging. All sections were examined under a Nikon Eclipse Ti-U confocal fluorescence microscope equipped with a digital camera (FV300, Olympus, Japan) [34, 35].

### Statistical analysis

All data are presented as mean ± standard deviation (SD). Normality was assessed using the Kolmogorov-Smirnov test, and homogeneity of variance was tested with Levene’s test. Comparisons between two groups were made using the independent samples t-test; comparisons among three or more groups were performed using one-way analysis of variance (ANOVA), with post hoc analysis conducted using Fisher’s least significant difference method. Data analysis was performed using SPSS Statistics 29.0, and graphs were generated using GraphPad Prism 8.0. A significance level of *p* < 0.05 was considered statistically significant.

## Results

### Alterations in hypothalamic rhythm-related hormones and gene expression under HH

**Fig. 1:**
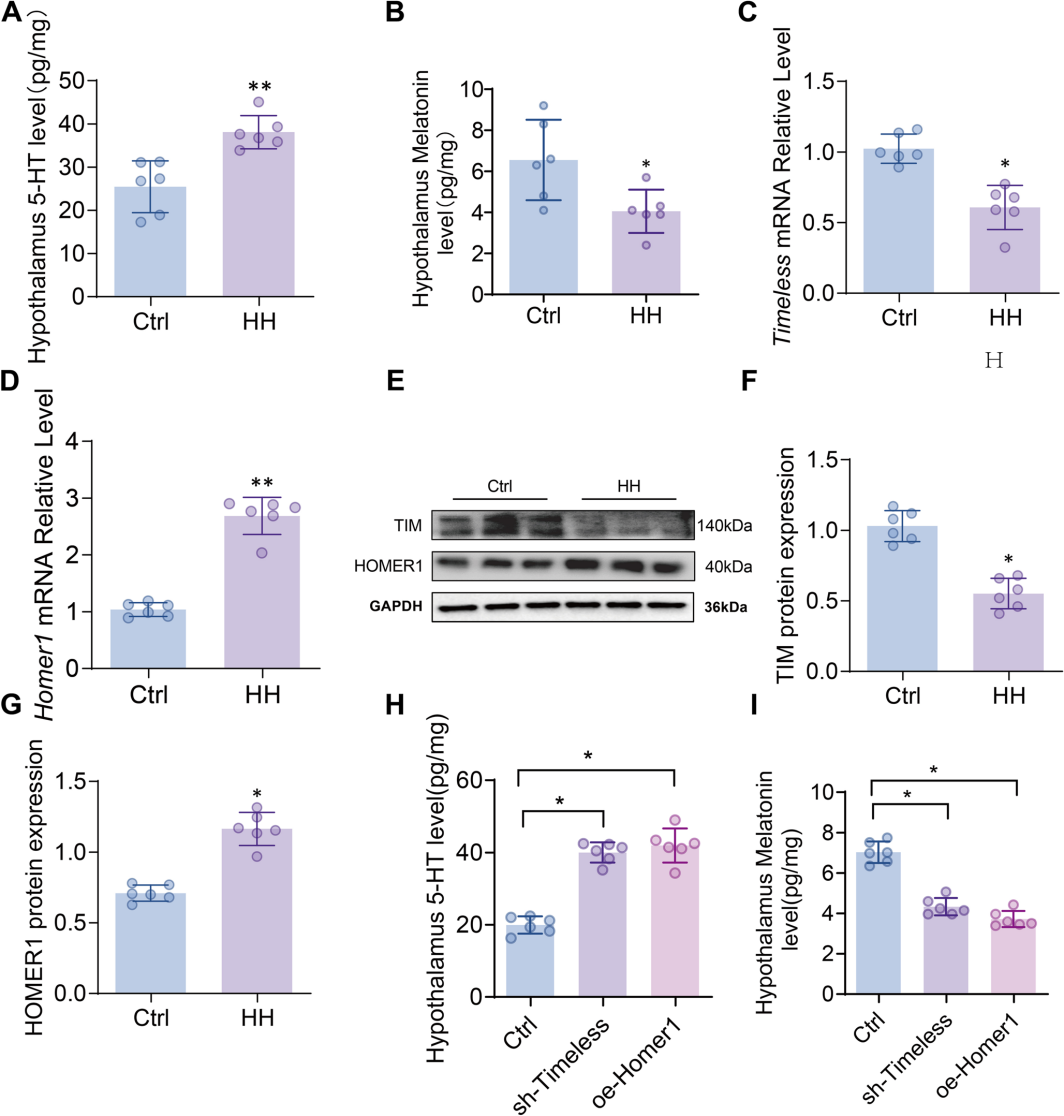
Effects of HH on Sleep Rhythm Hormones and Rhythm Gene Expression in the Rat Hypothalamus. (**A**) Levels of 5-HT in the hypothalamus (ELISA, *n* = 6); (**B**) Levels of melatonin in the hypothalamus (ELISA, *n* = 6); (**C**-**D**) Relative transcription levels of Timeless and Homer1 genes (RT-qPCR, *n* = 6); (**E**-**G**) Expression of TIM and HOMER1 proteins (WB densitometric analysis, *n* = 6); (**H**) Levels of 5-HT in the hypothalamus (ELISA, *n* = 6); (**I**) Levels of melatonin in the hypothalamus (ELISA, *n* = 6);. GAPDH was used as the internal reference. Data are presented as mean ± SD and statistical analysis was performed using a two-tailed Student’s t-test. *p < 0.05, **p < 0.01 vs. Ctrl, *n* = 6

In the HH group, hypothalamic 5-HT levels were significantly elevated (*p* < 0.01; Fig. 1A), while melatonin levels were significantly reduced compared with the Ctrl group (*p* < 0.05; Fig. 1B). The mRNA expression of Timeless was downregulated (*p* < 0.05; Fig. 1C), whereas Homer1 was upregulated (*p* < 0.01; Fig. 1D) in the HH group. Western blot analysis showed a corresponding decrease in TIM protein (*p* < 0.05; Fig. 1E and F) and an increase in HOMER1 protein (*p* < 0.05; Fig. 1E and G) relative to the Ctrl group. Compared with the control group, the sh-Timeless group and the oe-Homer1 group showed significantly increased 5-HT levels and significantly decreased melatonin levels (*p* < 0.05; Fig. 1H-I).

### HH reduces spontaneous activity and induces anxiety-like behavior in rats

**Fig. 2:**
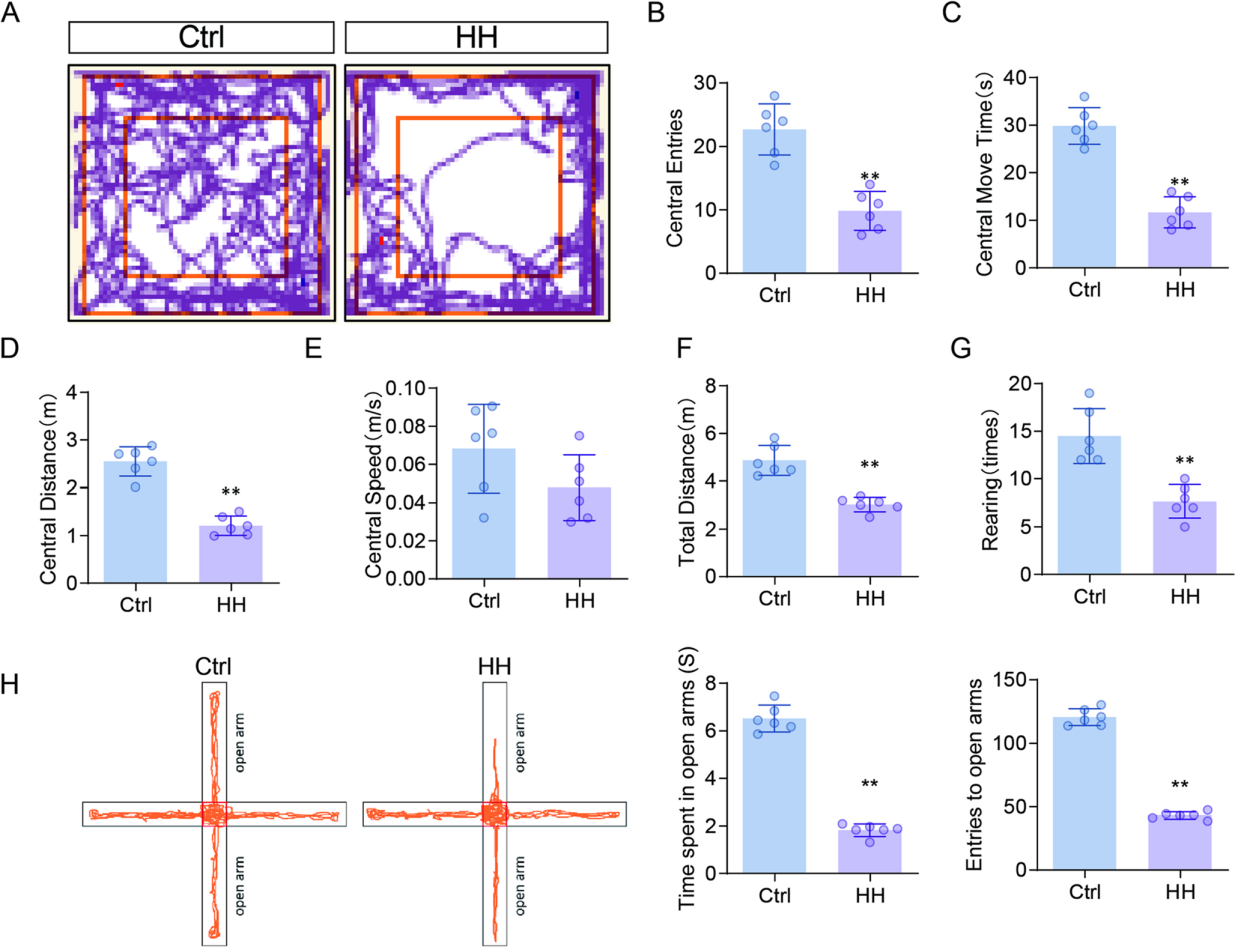
Effects of HH on Spontaneous Activity and Anxiety-like Behavior in Rats. (**A**) Trajectories from the OFT; (**B**) Number of entries into the central area; (**C**) Time spent in the central area; (**D**) Distance traveled in the central area; (**E**) Walking speed in the central area; (**F**) Total distance traveled; (**G**) Number of rearing events. (**H**) Open arm entries, time spent in open arms, and representative movement traces in the elevated plus maze. Data are presented as mean ± SD and statistical analysis was performed using a two-tailed Student’s t-test. **p < 0.01 vs. Ctrl, *n* = 6

In the open field test, rats in the HH group showed a significant reduction in the number of entries into the central area (*p* < 0.01; Fig. 2A-B), time spent in the center (*p* < 0.01; Fig. 2C), and distance traveled within the center (*p* < 0.01; Fig. 2D). A decreasing trend in center movement speed was also observed (Fig. 2E). Representative movement trajectories are shown in Fig. S2. For general locomotor activity, total distance traveled and rearing frequency were significantly lower in the HH group than in the Ctrl group (*p* < 0.01; Fig. 2F-G). In the elevated plus maze test, the HH group also exhibited a significant reduction in the number of entries into open arms and the time spent in open arms (*p* < 0.01; Fig. 2H).

### Dex reduces HH-induced elevation of inflammatory cytokines

**Fig. 3:**
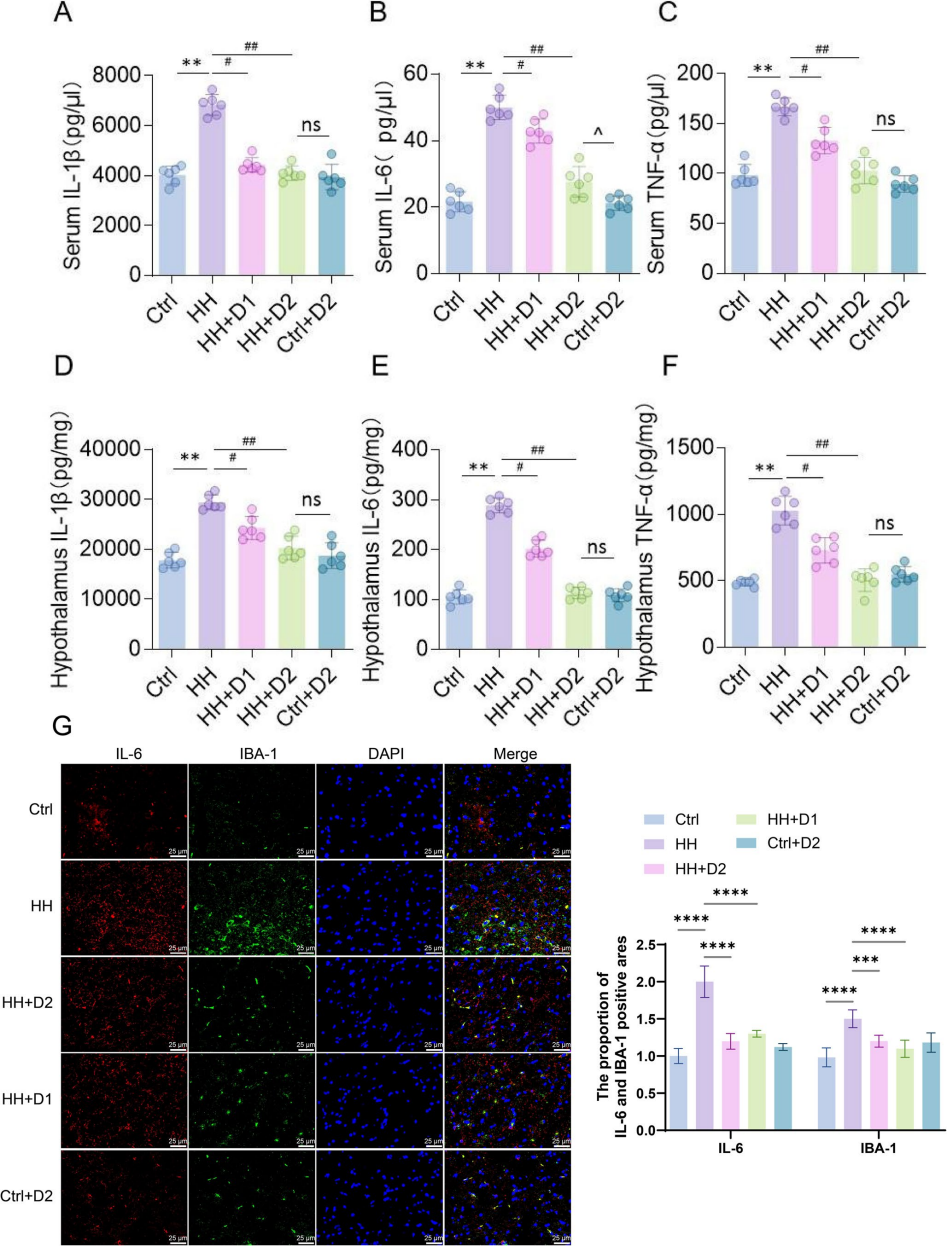
Effects of dexmedetomidine on inflammatory cytokine levels in serum and hypothalamus of rats exposed to high-altitude hypoxia. (**A**) Serum IL-1β levels; (**B**) serum IL-6 levels; (**C**) serum TNF-α levels; (**D**) hypothalamic IL-1β levels; (**E**) hypothalamic IL-6 levels; (**F**) hypothalamic TNF-α levels. (**G**) Representative immunofluorescence images and quantification showing co-localization of IL-6 and IBA-1 in brain sections. Data are presented as mean ± SD, *n* = 6 per group. Statistical analysis was performed using one-way ANOVA. *P* < 0.05, *P* < 0.01 vs. Ctrl; #*P* < 0.05, ##*P* < 0.01 vs. HH; *P* < 0.05 for Ctrl + D2 vs. HH + D2; ns, not significant

In serum, levels of IL-1β, IL-6, and TNF-α were significantly increased in the HH group compared to the Ctrl group (*p* < 0.01; Fig. 3A-C). Both low- and high-dose Dex treatments reduced these cytokine levels, with stronger suppression observed in the high-dose group (*p* < 0.01; Fig. 3A-C). No significant changes were detected in Dex-treated Ctrl animals. In the hypothalamus, IL-1β, IL-6, and TNF-α levels were also significantly elevated in the HH group (*p* < 0.01; Fig. 3D-F). Dex treatment reduced these levels in a dose-dependent manner, with greater reductions observed in the high-dose group (*p* < 0.05; Fig. 3D-F). Immunofluorescence staining showed enhanced co-expression of IL-6 and IBA-1 in the HH group, which was attenuated by Dex treatment in a dose-dependent manner (Fig. 3G).

### Activation of the TLR4-MyD88-NFκB pathway in the hypothalamus under HH

**Fig. 4:**
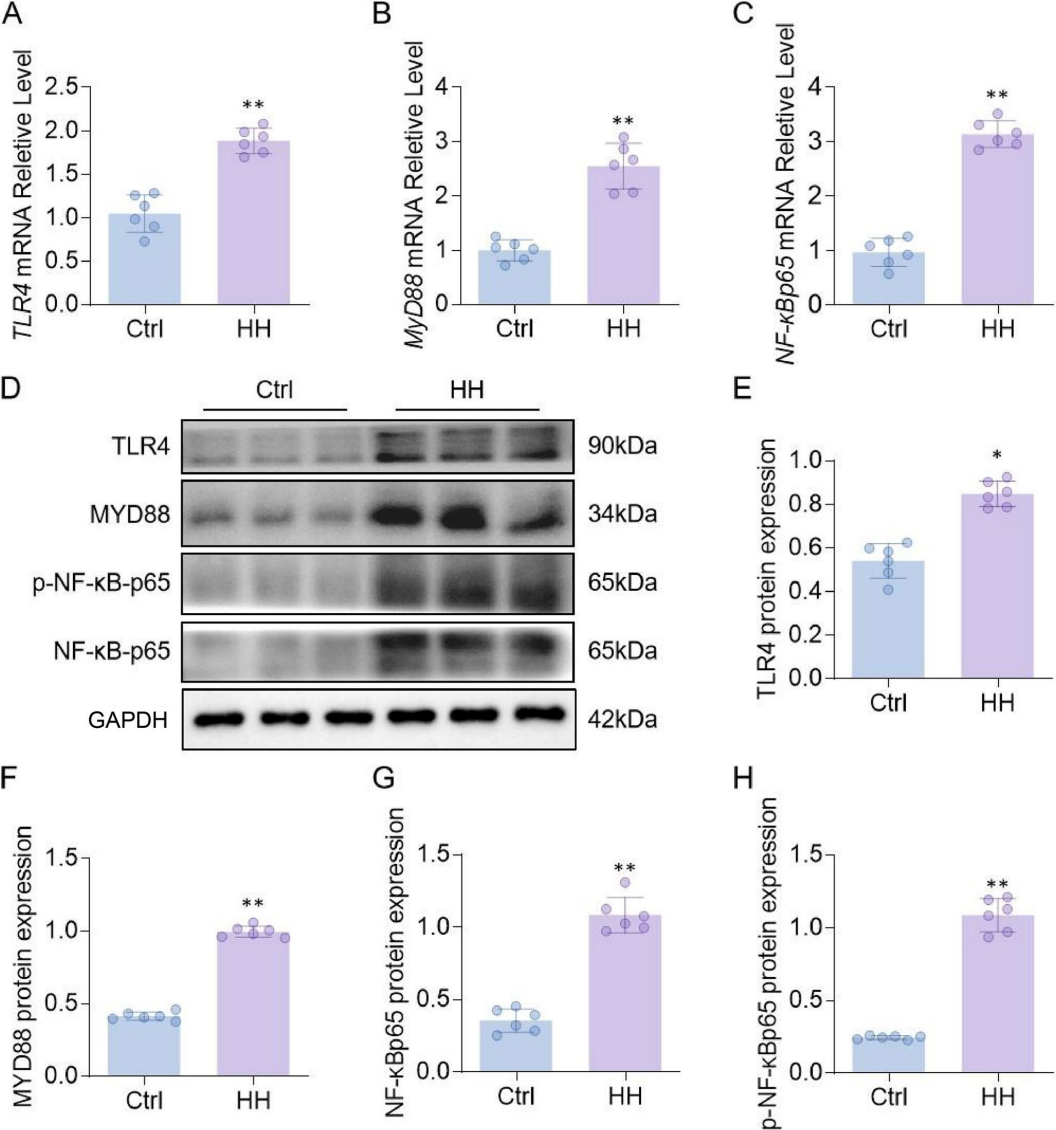
Effects of HH on Protein Levels in the TLR4-MyD88-NFκB Pathway in the Rat Hypothalamus. (**A**-**C**) Relative mRNA transcription levels of Tlr4, MyD88, and NF-κBp65 in the hypothalamus; (**D**) Protein expression of TLR4, MyD88, and NF-κBp65 detected by WB; (**E**-**H**) Densitometric analysis of the quantitative results. β-actin was used as the internal reference. Data are presented as mean ± SD and statistical analysis was performed using a two-tailed Student’s t-test. *p < 0.05, **p < 0.01 vs. Ctrl, *n* = 6

**Fig. 5:**
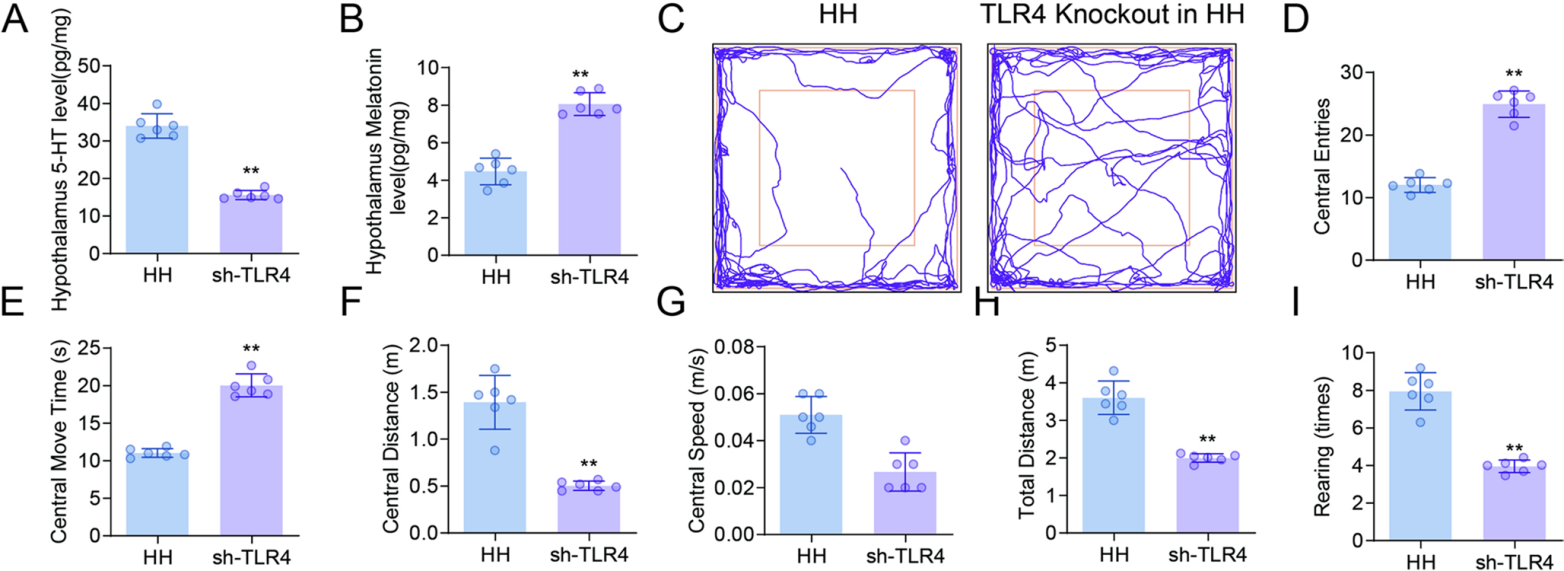
Knockdown of TLR4 Alleviates Hypothalamic Hormonal Dysregulation and Anxiety-like Behaviors Induced by High-Altitude Hypoxia in Rats. (**A**) Levels of 5-HT in the hypothalamus (ELISA, *n* = 6); (**B**) Levels of melatonin in the hypothalamus (ELISA, *n* = 6); (**C**) Trajectories from the OFT; (**D**) Number of entries into the central area; (**E**) Time spent in the central area; (**B**) Distance traveled in the central area; (**G**) Walking speed in the central area; (**H**) Total distance traveled; (**I**) Number of rearing events. Data are presented as mean ± SD and statistical analysis was performed using a two-tailed Student’s t-test. **p < 0.01 vs. Ctrl, *n* = 6

**Fig. 6:**
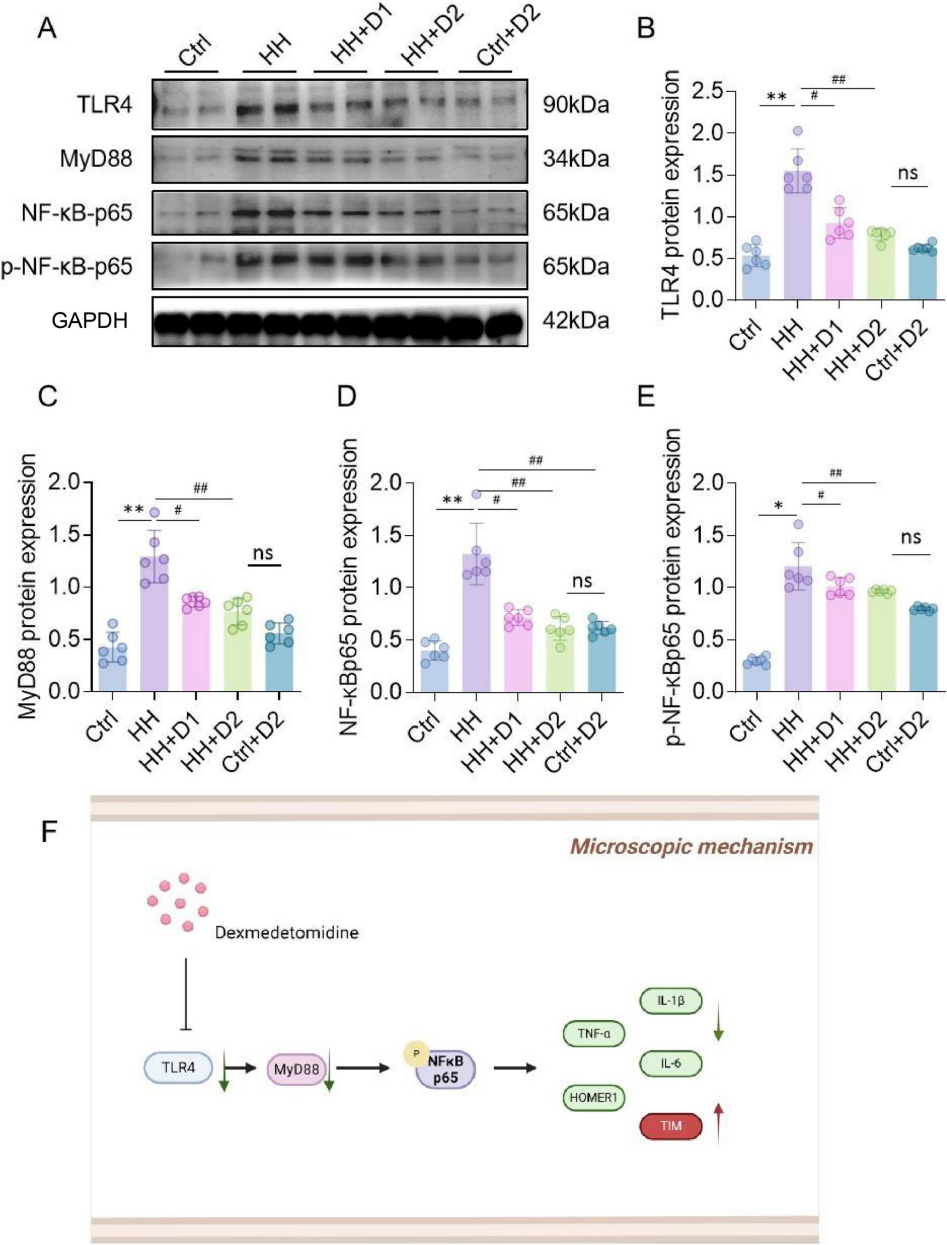
Effects of Dex on Protein Levels in the TLR4-MyD88-NFκB Pathway in the Hypothalamus of Rats Exposed to HH. (**A**) Expression levels of TLR4-MyD88-NFκB pathway proteins in the hypothalamus detected by WB; (**B**-**E**) Densitometric analysis results for TLR4, MyD88, NF-κBp65, and p-NF-κBp65; (**F**) Schematic diagram illustrating the mechanism by which Dex alleviates hypoxia-induced hypothalamic inflammation and behavioral abnormalities through the inhibition of the TLR4-MyD88-NFκB signaling pathway. Data are presented as mean ± SD and statistical analysis was performed using one-way ANOVA. *p < 0.05 vs. Ctrl; **p < 0.01; #*p* < 0.05 vs. HH; ##*p* < 0.01, *n* = 6. ns indicates no significant difference between groups

The HH group showed significantly elevated mRNA levels of Tlr4, MyD88, and NF-κBp65 in the hypothalamus compared with the Ctrl group (*p* < 0.05; Fig. 4A-C). Western blot analysis further confirmed increased protein expression of TLR4, MyD88, and NF-κBp65 in the HH group (*p* < 0.05; Fig. 4D-H). In the HH model, TLR4 knockdown rats (sh-TLR4) were generated to block activation of the TLR4-MyD88-NFκB pathway. TLR4 knockdown resulted in a significant decrease in hypothalamic 5-HT levels and a marked increase in melatonin levels compared with the HH group (Fig. 5A, B). Compared with HH rats, sh-TLR4 rats showed significantly increased numbers of center entries, longer time spent in the center, greater locomotor distance and higher velocity in the open field test (Fig. 5C-E). Total distance traveled and the number of rearings were also significantly elevated in the sh-TLR4 group (Fig. 5F, G). In the elevated plus maze, sh-TLR4 rats exhibited a significant increase in both open arm entries and time spent in the open arms, indicating reduced anxiety-like behavior (Fig. 5H, I). Compared with the HH group, protein expression levels of TLR4, MyD88, and NF-κBp65 in the hypothalamus were significantly decreased in the HH + Dex group (Fig. 6A-D). High-dose Dex treatment led to a downward trend in p-NF-κBp65 levels, although this difference did not reach statistical significance (Fig. 6A, E). A schematic overview of this signaling pathway is provided in Fig. 6F.

### Dex attenuates TLR4-MyD88-NFκB pathway activation under HH

Protein levels of TLR4, MyD88, and NF-κBp65 were significantly reduced in the HH + Dex group compared with the HH group (*p* < 0.05; Fig. 5A-D). A decreasing trend in p-NF-κBp65 expression was observed following high-dose Dex treatment, although the change was not statistically significant (Fig. 5A and E). Quantitative summary of the pathway is illustrated in Fig. 5F.

### Dex improves rhythm-related hormonal and gene expression alterations under HH

**Fig. 7:**
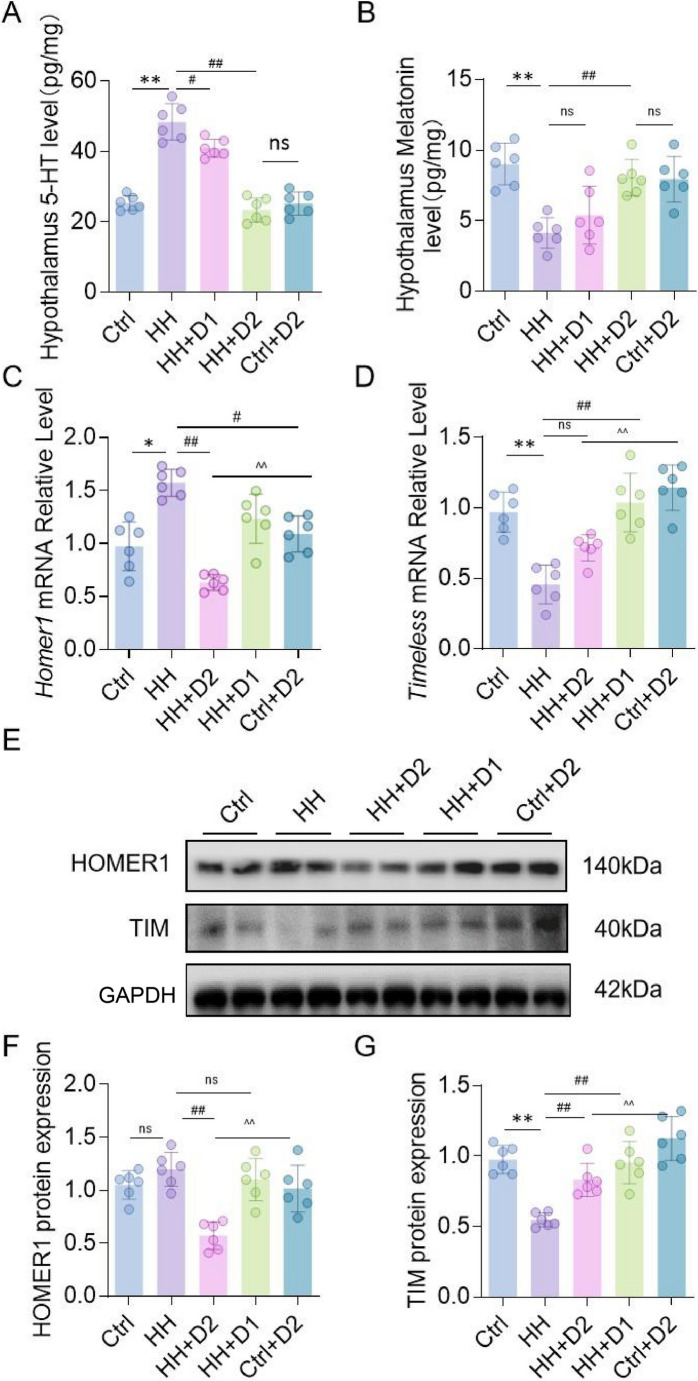
Effects of Dex on Sleep Rhythm-Related Molecules in the Hypothalamus of Rats Exposed to HH. (**A**-**B**) Levels of 5-HT and melatonin in the hypothalamus; (**C**-**D**) Relative transcription levels of Homer1 and Timeless genes; (**E**-**G**) Expression levels of HOMER1 and TIM proteins and densitometric analysis. Data are presented as mean ± SD and statistical analysis was performed using one-way ANOVA. *p < 0.05 vs. Ctrl; **p < 0.01; #*p* < 0.05 vs. HH; ##*p* < 0.01; ^*p* < 0.05 vs. Ctrl + D2 vs. HH + D2, ^^*p* < 0.01, *n* = 6. ns indicates no significant difference between groups

Compared to the Ctrl group, the HH group exhibited significantly increased 5-HT and decreased melatonin levels in the hypothalamus (*p* < 0.01; Figs. 6B and 7A). Dex treatment reduced 5-HT and restored melatonin levels, with greater effects observed in the high-dose group (*p* < 0.01). At the mRNA level, HH-induced upregulation of Homer1 was significantly reduced by high-dose Dex (*p* < 0.01; Fig. 7C), while Timeless transcription was significantly increased by low-dose Dex compared with the HH group (*p* < 0.05; Fig. 7D). For protein expression, HH elevated HOMER1 and reduced TIM levels; high-dose Dex significantly downregulated HOMER1 and upregulated TIM protein expression (*p* < 0.05; Fig. 7E-G).

### Dex improves locomotor activity and anxiety-like behavior in rats under HH

**Fig. 8:**
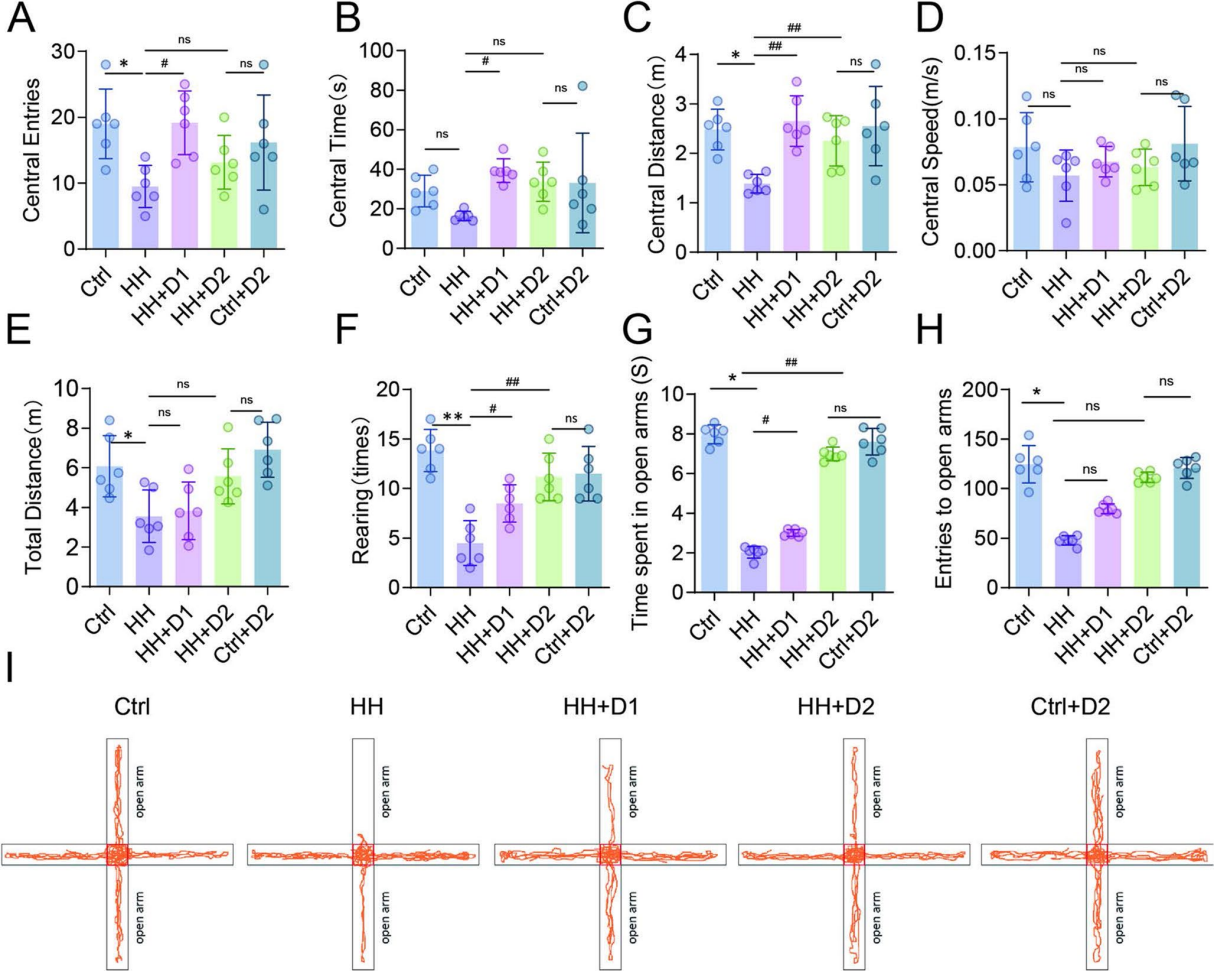
Effects of Dex on Spontaneous Activity and Anxiety-like Behavior in Rats Exposed to HH. (**A**-**F**) Open field test: (**A**) number of center entries; (**B**) time spent in center; (**C**) distance traveled in center; (**D**) movement speed in center; (**E**) total distance traveled; (**F**) number of rearings. (**G**-**H**) Elevated plus maze: (**G**) time in open arms; (**H**) number of open arm entries. (**I**) Representative movement traces in the elevated plus maze. Data are presented as mean ± SD and statistical analysis was performed using one-way ANOVA. *p < 0.05 vs. Ctrl; **p < 0.01; #*p* < 0.05 vs. HH; ##*p* < 0.01, *n* = 6. ns indicates no significant difference between groups Graphic abstract Mechanism by Which Dex Alleviates HH-Induced Neuroinflammation and Behavioral Abnormalities by Inhibiting the TLR4-MyD88-NFκB Pathway

The HH group showed significantly reduced number of entries, time spent, and distance traveled in the center zone compared to the Ctrl group (*p* < 0.01; Fig. 8A-C). Dex treatment reversed these effects, with high-dose Dex producing more pronounced improvements (*p* < 0.01). Low-dose Dex also significantly increased center entries and distance (*p* < 0.05; Fig. 8A-C). HH exposure led to reduced movement speed in the center zone (*p* < 0.01), which was significantly improved by both Dex doses, especially the high dose (*p* < 0.01; Fig. 8D). Dex also increased total distance traveled (*p* < 0.01; Fig. 8E) and number of rearings (*p* < 0.05; Fig. 8F), with a more prominent effect on distance observed in the high-dose group, while both doses similarly improved rearing frequency. In the elevated plus maze test, Dex significantly increased the number of open arm entries and time spent in open arms (*p* < 0.05; Fig. 8G-I).

## Discussion

HH has been shown to disrupt sleep rhythm, emotional stability, and neuroimmune homeostasis in both humans and animal models [36–38]. However, the underlying mechanisms Linking hypoxic exposure to these physiological disturbances remain insufficiently defined. In this study, sustained HH exposure induced significant alterations in hypothalamic 5-HT and melatonin levels, accompanied by changes in the expression of rhythm-related genes Timeless and Homer1. These molecular changes were associated with impaired spontaneous activity, heightened anxiety-like behavior, and increased neuroinflammatory responses. Together, these findings highlight the hypothalamus as a key integrative center mediating hypoxia-induced disruptions in neuroendocrine and behavioral rhythms, providing new insights into the pathophysiology of high-altitude environments.

Chronic HH exposure increased 5-HT and decreased melatonin levels in the hypothalamus. This contrasts with findings from acute hypoxia models, which often report reduced 5-HT release or content, particularly in the prefrontal cortex and hippocampus [5]. The discrepancy May reflect differences in hypoxia duration, model type, or brain region examined. The hypothalamus, as a central regulator of circadian and neuroendocrine signaling, May respond to hypoxia with compensatory neurotransmitter adaptation. Elevated peripheral 5-HT levels have also been reported in high-altitude-adapted populations [39], and increased 5-HT terminal density has been observed in selective brain regions under chronic intermittent hypoxia [40]. These findings suggest that serotonergic plasticity may be involved in the systemic adaptation to sustained low oxygen exposure. In contrast, the decline in melatonin is consistent with prior studies and may reflect direct circadian disruption by hypoxia [33]. Together, these changes in hypothalamic 5-HT and melatonin may contribute to sleep rhythm disturbances induced by HH.

This study demonstrated that prolonged HH significantly downregulated the transcription of the circadian gene Timeless, while inducing moderate changes in Homer1. Timeless, a core component of the molecular clock, has been widely implicated in maintaining circadian rhythmicity, and its suppression under hypoxic conditions may reflect circadian reprogramming or disruption of clock stability [41]. Homer1, which regulates synaptic signaling and plasticity, also exhibited altered expression, suggesting that sustained hypoxia may interfere with neuronal communication. Knockdown of Timeless and overexpression of Homer1 both resulted in increased 5-HT and decreased melatonin levels, indicating that dysregulation of these circadian genes can directly induce hypothalamic hormonal changes. These findings extended previous research by identifying specific gene-level disruptions in the hypothalamus, thereby linking hypoxia-induced molecular changes to impaired rhythm regulation and behavioral outputs.

Behavioral analysis using the OFT revealed that rats exposed to HH exhibited reduced spontaneous activity and increased anxiety-like behavior. These results were consistent with earlier reports showing that chronic hypoxia induces anxiety-like phenotypes and psychomotor impairments, possibly through inflammation and neurotransmitter imbalance. Previous studies have associated such behavioral alterations with dysfunction of serotonergic and GABAergic systems [5, 42]. The present findings suggested that HH May indirectly shape emotional and behavioral responses by modulating hypothalamic levels of 5-HT and melatonin. Compared with acute hypoxia models, the use of a sustained hypobaric hypoxia model in this study provided new insights into the long-term behavioral consequences of environmental hypoxia.

HH exposure induced neuroinflammatory responses in the hypothalamus, as evidenced by increased microglial activation and elevated expression of IL-1β, IL-6, and TNF-α, alongside upregulation of the TLR4-MyD88-NFκB signaling pathway. These findings were consistent with previous studies reporting that hypoxia triggers central inflammation via this pathway [43]. Although this study did not directly assess HIF-1α, prior work has demonstrated that HIF-1α can mediate immune activation under hypoxic conditions through TLR4-MyD88-NFκB signaling [44]. In addition, melatonin has been shown to attenuate hypoxia-induced inflammation by reducing HIF-1α stabilization [45]. The observed reduction in melatonin, coinciding with enhanced inflammatory signaling, may reflect this regulatory axis. Treatment with dex significantly reduced the expression of TLR4, MyD88, and NFκBp65 in HH-exposed rats and alleviated neuroinflammatory responses and anxiety-like behaviors. These results suggested that Dex may exert its anti-inflammatory effects through inhibition of the TLR4-MyD88-NFκB pathway. However, this mechanism was not directly validated using specific inhibitors such as TAK-242. Moreover, alternative pathways including HIF-1α and NLRP3 may also contribute to the observed effects [46, 47]. Further studies using pharmacological or genetic approaches are needed to clarify the specific mechanisms involved.

This study revealed the mechanisms by which HH affected physiological functions such as sleep rhythm, neuroinflammation, and anxiety-like behavior, providing a theoretical basis for the treatment of related disorders, including high-altitude sickness, sleep disturbances, and anxiety. Notably, the intervention effects of dex on hypoxia-induced dysfunctions supported its potential for clinical application. Given its anti-inflammatory properties, dex represented a promising candidate for managing hypoxia-associated conditions. Furthermore, the findings offered valuable insight for future research into other pharmacological or therapeutic strategies, such as anti-inflammatory agents and sleep modulators.

This study had several limitations. First, it was based on an animal model, and although the findings provided theoretical insights, clinical validation remains necessary. In addition, only male rats were used in this study. Given the known influence of sex hormones on sleep, neuroinflammation, and drug metabolism [33], sex differences may significantly affect the outcomes, and the generalizability of these findings to females remains uncertain. Future studies should include both male and female animals to assess sex-dependent responses.In addition, the absence of polysomnographic recordings limited the comprehensive assessment of sleep-wake rhythms in rats. The Dex dosage and administration schedule used in this study also differed from clinical practice, necessitating further optimization to improve translational relevance. Sequential assessments were not performed, making it difficult to capture the dynamic progression of hypoxic effects and the temporal profile of Dex intervention. Future studies will incorporate time-course evaluations to systematically characterize the evolution of hypoxic stress and the timing of Dex effects. Moreover, the study did not include active comparators such as melatonin or GABAergic hypnotics (e.g., zolpidem), Limiting the assessment of the specificity and comparative efficacy of dex in the context of hypoxia-induced circadian disruption. Finally, although changes in 5-HT and melatonin levels were observed alongside alterations in the expression of rhythm-related genes, the underlying regulatory relationships remain unclear. Future studies should incorporate pharmacological or genetic tools to elucidate causal mechanisms.

Further investigation is required to evaluate the translational potential of dex in clinical settings, particularly for managing altitude-related physiological disturbances. Studies examining dose-response relationships, alternative administration routes, and combination therapies with anti-inflammatory or circadian-modulating agents will provide a broader basis for therapeutic application. In addition, expanding the mechanistic scope to include interactions among neural, immune, and endocrine systems under hypoxic conditions could yield a more comprehensive understanding of high-altitude pathophysiology. Integrating pharmacological, neuroscientific, and clinical approaches may facilitate the development of targeted interventions for hypoxia-induced dysfunction.

## Conclusion

This study demonstrated that HH disrupts multiple physiological systems in rats, notably sleep rhythm, neuroinflammation, anxiety-like behavior, and locomotor activity. HH elevated hypothalamic 5-HT and reduced melatonin levels, accompanied by altered expression of the rhythm-related genes Timeless and Homer1. These changes were associated with increased levels of IL-1β, IL-6, and TNF-α and behavioral deficits. Dex effectively attenuated these abnormalities by suppressing inflammatory responses, restoring hormone balance, and improving behavioral outcomes. These findings support its potential therapeutic value in managing hypoxia-induced dysfunctions. Further studies are needed to clarify the causal link between hormone levels and clock gene expression and to compare Dex with standard chronotherapeutics to refine intervention strategies under hypoxic conditions.

## Supplementary Information

Below is the link to the electronic supplementary material.

Supplementary Material 1. Fig. 1Schematic Diagram of Experimental Grouping and Treatment Protocol (JPG 380 KB)

Supplementary Material 2. Fig. 2Trajectories of Different Groups of Rats in the OFT (JPG183 KB)

Supplementary Material 3 (DOCX 16.5 KB)

Supplementary Material 4 (JPG317 KB)

Supplementary Material 5 (JPG571 KB)

Supplementary Material 6 (JPG416 KB)

Supplementary Material 7 (JPG 343 KB)

## Data Availability

All data can be provided as needed.

## Abbreviations

5-HT: 5-hydroxytryptamine
ANOVA: Analysis of Variance
Dex: Dexmedetomidine
HH: High-altitude Hypoxia
IL-1β: Interleukin-1 beta
IL-6: Interleukin-6
OFT: Open Field Test
OD: Optical Density
RT-qPCR: Reverse Transcription Quantitative PCR
SD: Standard Deviation
TNF-α: Tumor Necrosis Factor-alpha
WB: Western Blot
