# Supplementary figures and images for: Dexmedetomidine regulates sleep rhythm and alleviates neuroinflammation in rats under high-altitude hypoxia
Source: J Physiol Biochem. 2025 Nov 12;81(4):1125–41. doi: 10.1007/s13105-025-01127-1 (PMC12738677; doi:10.1007/s13105-025-01127-1)

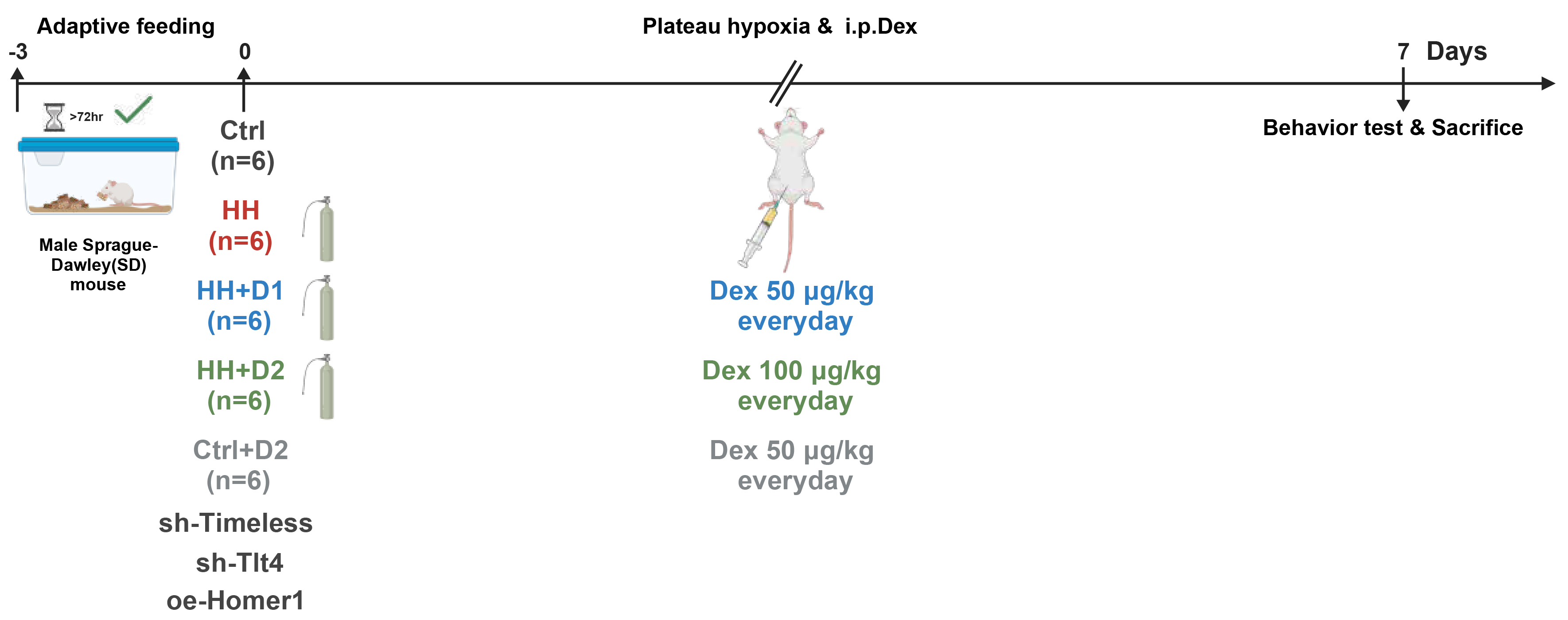

Supplement: Supplementary file 1 — Supplementary Material 1. Fig. 1Schematic Diagram of Experimental Grouping and Treatment Protocol (JPG 380 KB) [file 13105_2025_1127_MOESM1_ESM.jpg]

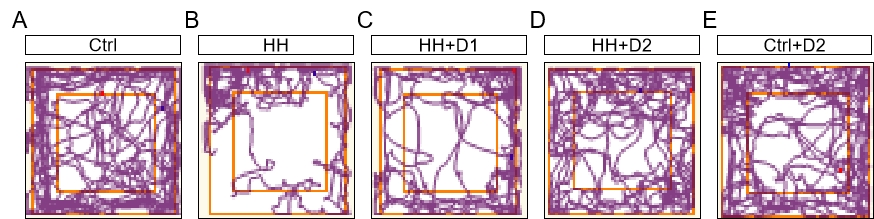

Supplement: Supplementary file 2 — Supplementary Material 2. Fig. 2Trajectories of Different Groups of Rats in the OFT (JPG183 KB) [file 13105_2025_1127_MOESM2_ESM.jpg]

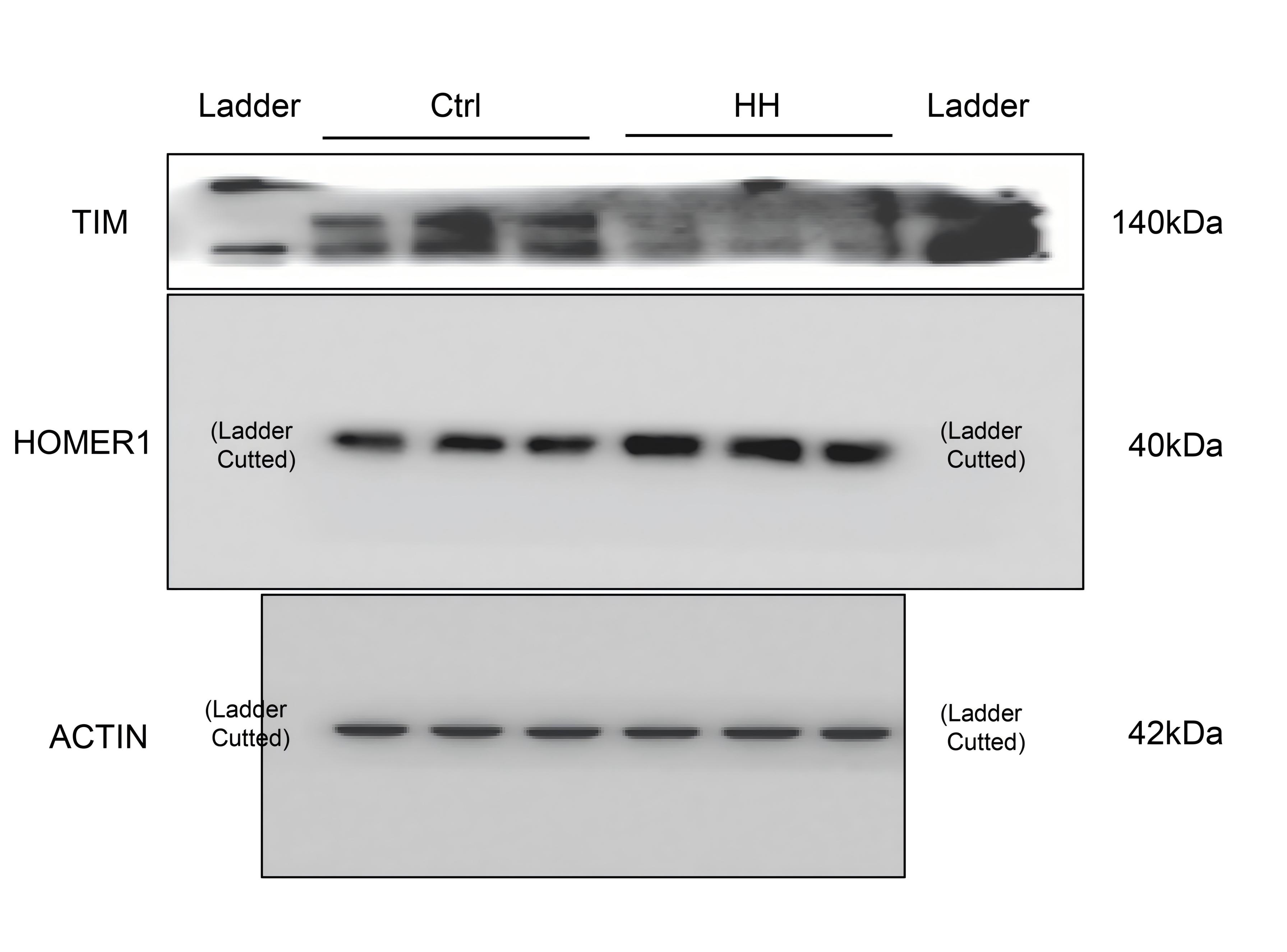

Supplement: Supplementary file 4 — Supplementary Material 4 (JPG317 KB) [file 13105_2025_1127_MOESM4_ESM.jpg]

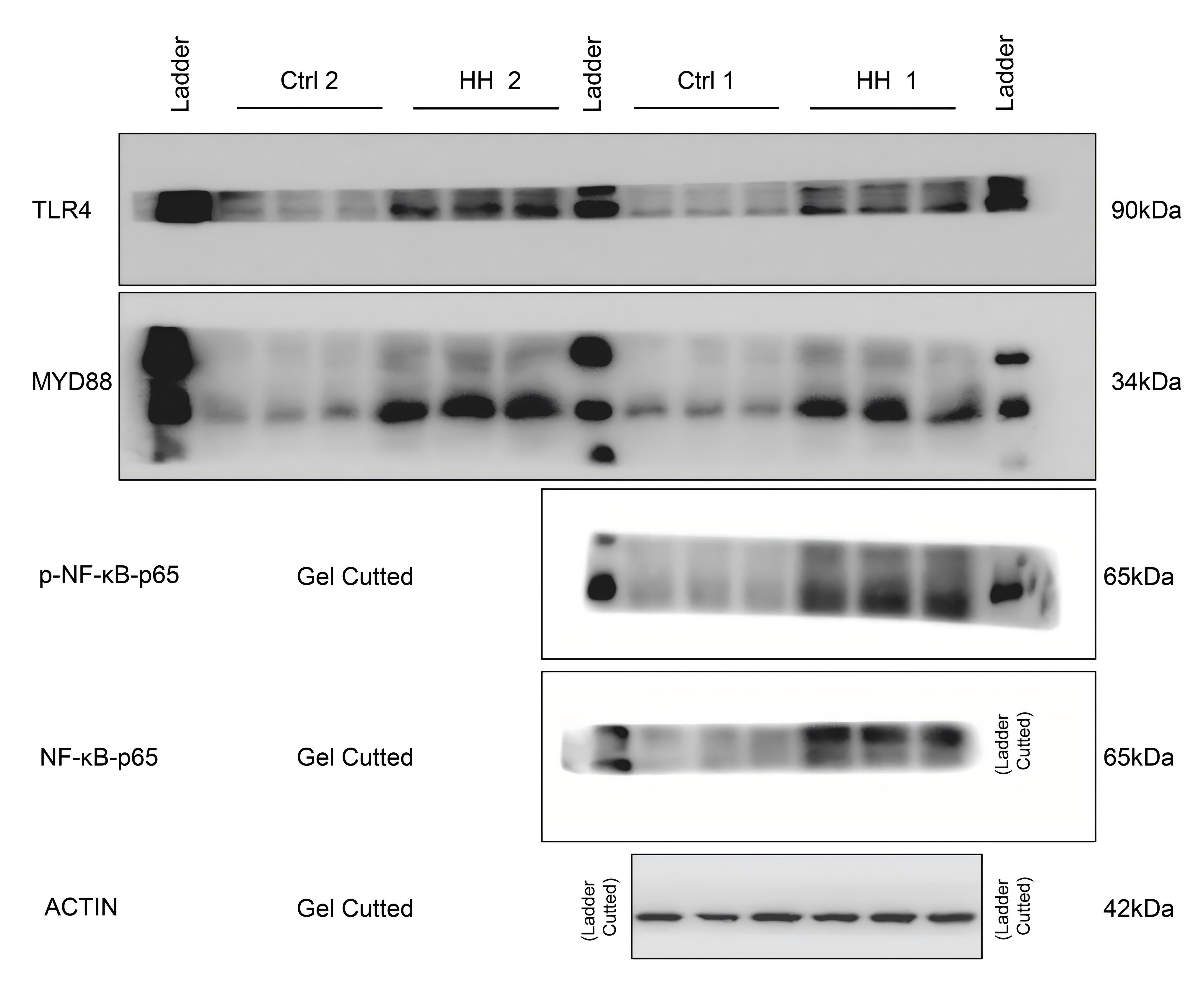

Supplement: Supplementary file 5 — Supplementary Material 5 (JPG571 KB) [file 13105_2025_1127_MOESM5_ESM.jpg]

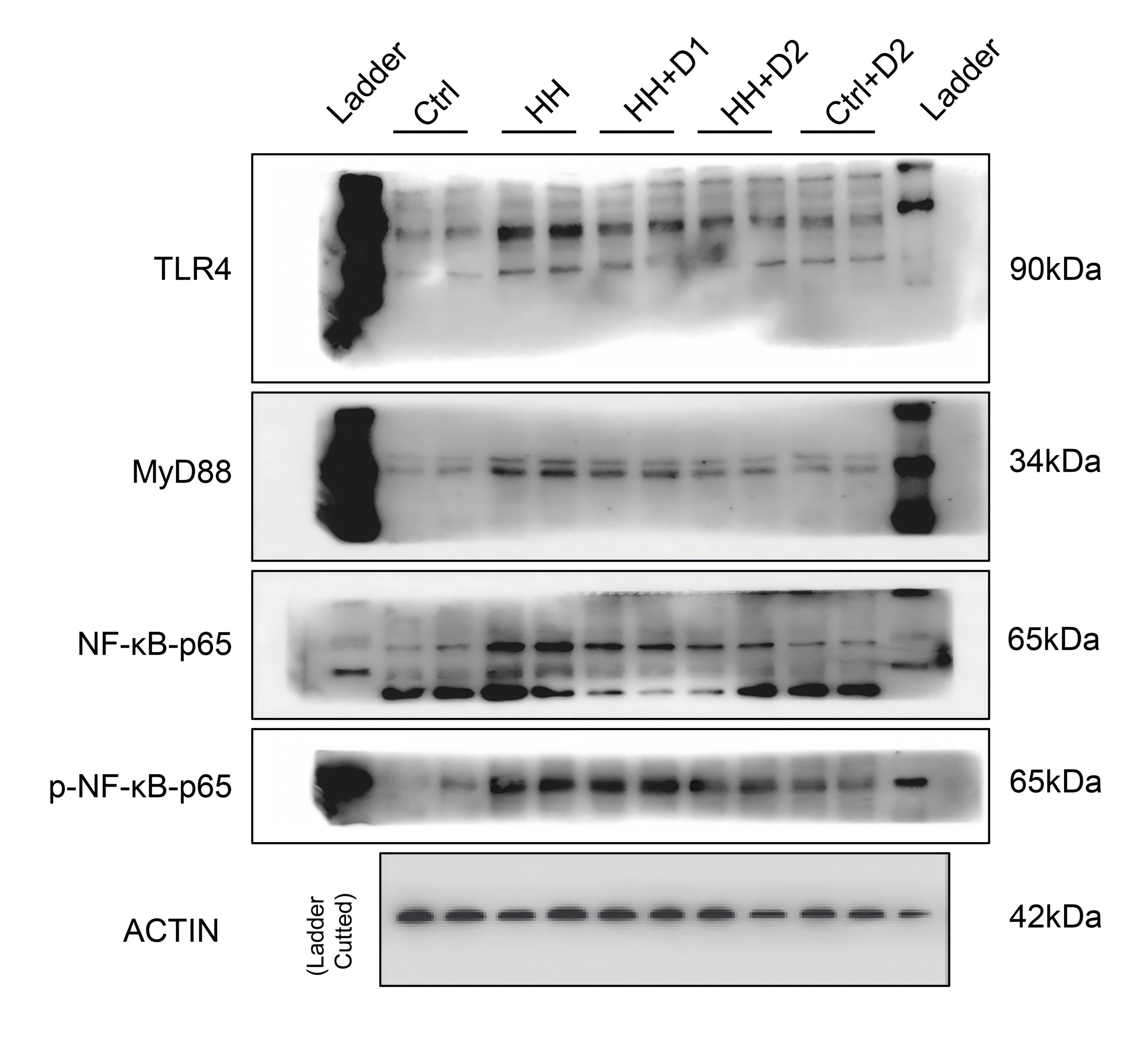

Supplement: Supplementary file 6 — Supplementary Material 6 (JPG416 KB) [file 13105_2025_1127_MOESM6_ESM.jpg]

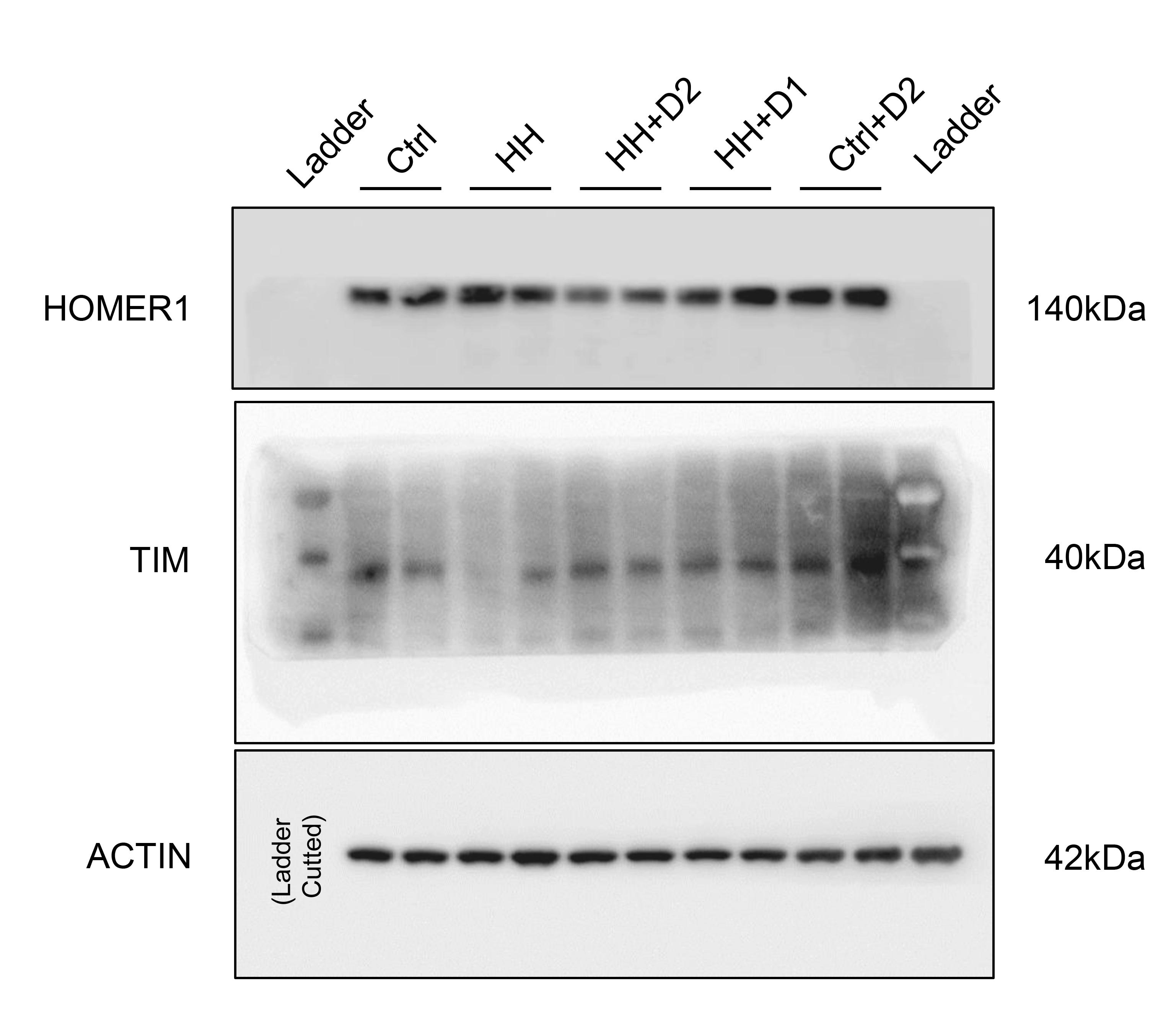

Supplement: Supplementary file 7 — Supplementary Material 7 (JPG 343 KB) [file 13105_2025_1127_MOESM7_ESM.jpg]
