# Supplementary material for: Dexmedetomidine regulates sleep rhythm and alleviates neuroinflammation in rats under high-altitude hypoxia
Source: J Physiol Biochem. 2025 Nov 12;81(4):1125–41. doi: 10.1007/s13105-025-01127-1 (PMC12738677; doi:10.1007/s13105-025-01127-1)
Supplement: Supplementary file 3 — Supplementary Material 3 (DOCX 16.5 KB) [file 13105_2025_1127_MOESM3_ESM.docx]

**Table S1 Nucleotide sequences of the primers used for RT-qPCR.**

| **Gene** | **Primer Sequence (5'→3')** | **PCR Product(bp)** |
| --- | --- | --- |
| [TIMELESS](https://www.ncbi.nlm.nih.gov/gene/8914) | 5'-GCCCTCAATGTGAGGCTCTT-3'(F)  5'-CCCGAAGCAGGTGATCCTTT-3'(R) | 100 |
| Homer1 | 5'- CCCTCTCTCATGCTAGTTCAGC -3'(F)  5'- GCACAGCGTTTGCTTGACT -3'(R) | 141 |
| TLR4 | 5'- ATGGCATGGCTTACACCACC-3'(F)  5'- GAGGCCAATTTTGTCTCCACA-3'(R) | 141 |
| MYD88 | 5'- ATCGCTGTTCTTGAACCCTCG-3’(F)  5'- CTCACGGTCTAACAAGGCCAG-3'(R) | 80 |
| NF-κB p65 | 5'- ACTGCCGGGATGGCTACTAT -3’(F)  5'- TCTGGATTCGCTGGCTAATGG -3'(R) | 141 |
| GAPDH | 5'- AGTGCCAGCCTCGTCTCATA-3'(F)  5'- GATGGTGATGGGTTTCCCGT-3'(R) | 150 |
